# Low density lipoprotein receptor-related protein 8: a critical receptor for tick-borne encephalitis virus entry

**DOI:** 10.1038/s41392-025-02509-z

**Published:** 2025-12-30

**Authors:** Pengtao Jiao, Lin Du, Yan Zeng

**Affiliations:** 1 Institute of Animal Science, Chinese Academy of Agricultural Sciences, Beijing, China; 2 School of Life Sciences, Tianjin University, Tianjin, China

**Keywords:** Cell biology, Microbiology

Recently, two groups identified low-density lipoprotein receptor-related protein 8 (LRP8) as a functional entry receptor for tick-borne encephalitis virus (TBEV) in *Nature*^1^ and *PNAS*^2^ respectively. These two studies shed crucial light on the role of LRP8 in the neuropathogenesis of TBEV and lay a solid foundation for the development of LRP8-targeted antiviral therapeutics and vaccines.

Tick-borne encephalitis virus (TBEV) is a clinically significant orthoflavivirus transmitted to humans predominantly via the bite of infected ticks. It targets the central nervous system (CNS), leading to neurological complications and potentially fatal outcomes in severe cases across Europe and Asia.^3^ With the expansion of TBE-endemic areas, vaccines are available, but no specific antiviral drugs exist for TBEV infection, highlighting the urgent need for in-depth research into TBEV pathogenic mechanisms and the development of effective preventive and therapeutic strategies.^4^ TBEV entry into host cells relies on host factors, particularly cellular receptors, which have not been definitively identified before, hindering the advancement of antiviral strategies.

Mittler et al.^1^ firstly conducted a comprehensive genome-wide CRISPR-Cas9 knockout screening of 21,913 genes in human A549 cells (a human lung adenocarcinoma cell line), systematically identifying LRP8 as a key host factor for TBEV infection. LRP8-knockout cell lines generated via CRISPR-Cas9 technology presented a greater than 50% reduction in TBEV reporter virus infectivity and a greater than 98% decrease in susceptibility across different authentic TBEV subtypes. In contrast, LRP8 overexpression increased TBEV reporter virus infectivity by approximately 2.3-fold compared with that in wild-type cells, whereas susceptibility to the European TBEV strain and Far Eastern TBEV subtype increased by over 8-fold and 4-fold, respectively. Notably, the reintroduction of LRP8 expression in knockout cells restored TBEV infectivity. Among the 13 LDLR homologs (including LDLR, VLDLR, LRP1, LRP2, LRP3, LRP4, LRP5, LRP6, LRP8, LRP10, LRP11, LRP12, and LRPAP1), only the knockdown or knockout of LRP8 effectively inhibited viral infection. Importantly, this dependence appears to be specific to TBEV but not to other tested orthoflaviviruses, including Japanese encephalitis virus, West Nile virus, Zika virus, and dengue virus, indicating a unique virus‒receptor interaction. Mechanistic mapping revealed that LRP8 directly interacts with the TBEV E glycoprotein via its ligand-binding domain. Systematic truncation analyses identified LA1-2 as the minimal functional unit mediating viral entry, whereas LA1-3 retained full activity, whereas LA1 alone exhibited only partial function. The viral binding site is localized to domain III (DIII) of the E protein, and stable interactions require multivalent presentation via a pentameric scaffold (COMP), mimicking the virion surface environment. This configuration enabled high-avidity interactions, as confirmed by coimmunoprecipitation, ELISA, and microscale thermophoresis. Functionally, LRP8 facilitates both viral attachment to host cells and subsequent internalization via the endocytic pathway (Fig. 1a). A translationally significant finding was the development of a soluble LRP8(LA1-2)-Fc decoy protein, which exhibited potent antiviral activity across multiple human cell types, including hepatoma, glioblastoma, and induced pluripotent stem cell-derived neuronal cultures. In vivo validation in a lethal mouse challenge model revealed robust protection. Compared with uniform mortality, administration via preincubation with virus or prophylaxis 6 h before infection reduced the brain viral load by more than 99.9%, minimized neuropathology, and achieved 95% survival (19/20 mice) in the control group. These results confirm that targeting the LRP8-TBEV E interaction effectively inhibits TBEV neural invasion and pathogenic processes in vivo. These findings indicate that strategies targeting the LRP8-TBEV E interaction can effectively inhibit TBEV neural invasion and pathogenic processes in vivo.

**Fig. 1:**
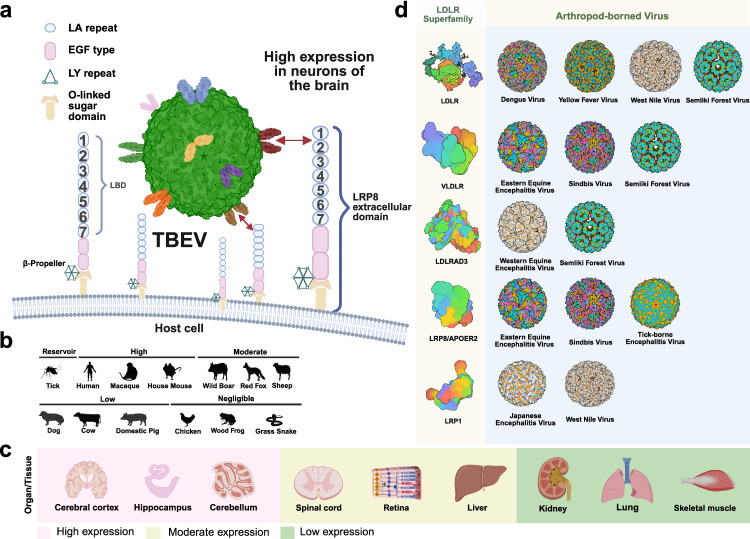
Schematic illustration of the viral characteristics of TBEV and its interaction with the host factor LRP8. **a** The binding mechanism of TBEV E DIII to LRP8 is depicted, highlighting the specific regions involved in the interaction and the role of LRP8 in viral entry. The TBEV virion structure is shown, emphasizing the high expression of LRP8 in neurons of the brain and its role in viral attachment and internalization. **b** TBEV susceptibility profiles of vertebrates and tick vectors. Mammalians exhibit different viraemia and mortality, whereas birds, amphibians, and reptiles are largely negligible hosts. Ticks serve as the true reservoir. **c** Correlation between TBEV neurotropism and the abundance of LRP8 across human tissues. The increase in the level of the LRP8 protein parallels viral neurotropism, with the highest levels in the cerebral cortex, hippocampus, and cerebellum. **d** The LRP8 receptor is shown in the context of the LDLR superfamily. Some important arthropod-borne viruses that utilize receptors from the LDLR superfamily for entry are also listed. The figure was created with BioRender.com

Li et al.^2^ adopted a targeted screening strategy based on functional speculation of LDLR receptor family members to identify the entry receptor of TBEV. By ectopically expressing the ligand-binding domains (LBDs) of 13 selected human LDLR family members in K562 cells (an erythroleukemic cell line with low susceptibility to orthoflaviviruses), they identified LRP8 as a candidate entry receptor for TBEV strains covering all five subtypes of the genus. While the two teams used distinct methodologies to identify LRP8 as a critical entry receptor for TBEV, both independently pinpointed the LA1-2 ligand-binding domain of LRP8 as the key region mediating interaction with the viral E protein. Li et al. further revealed that the receptor function of LRP8 is not limited to TBEV but extends to several related viruses in the TBE serocomplex, including Omsk hemorrhagic fever virus (OHFV), Louping ill virus (LIV), Alkhu rma virus (ALKV), and Kyasanur Forest disease virus (KFDV). These findings provide new insights into the common invasion mechanism of these tick-borne viruses. Analysis of LRP8 homologs in other mammalian species demonstrated functional conservation across different mammalian hosts, which may contribute to the broad host range of TBEV. Moreover, using primary neuronal cultures derived from LRP8 knockout mice, they directly demonstrated strong inhibition of TBEV infection upon LRP8 deletion in a physiologically relevant system, providing genetic evidence for the physiological importance of this receptor function.

In summary, these complementary studies confirm that LRP8 is a functional entry receptor for TBEV and related tick-borne orthoflaviviruses. Mittler’s genome-wide screen identified LRP8 as a specific receptor among LDLR family members, whereas Li’s targeted approach demonstrated its ability to mediate infection across TBEV subtypes and related viruses. Both studies convergently identified the LA1-2 domains of LRP8 as the critical interface for viral attachment, providing a mechanistic basis for TBEV neurotropism and laying the groundwork for therapeutic interventions. Notably, ticks, but not mammals, birds, amphibians, or reptiles, constitute the major reservoir for TBEV (Fig. 1b), and LRP8 is highly expressed in the human cerebral cortex (Fig. 1c). However, several limitations require attention. First, while soluble LRP8 decoys show prophylactic efficacy, genetic evidence from conditional LRP8 knockout models is needed to confirm its physiological role in natural infection. Second, the therapeutic potential of LRP8-based strategies remains unvalidated, as neither study assessed postexposure treatment efficacy against established CNS infection. Third, the structural basis of the LRP8-TBEV interaction requires elucidation through high-resolution complex analysis to identify key binding residues. Finally, investigation of LRP8 homologs in tick vectors and reservoir hosts will illuminate viral ecology and identify targets for transmission-blocking interventions. Future studies should address whether other LDLR members serve as receptors for additional orthoflaviviruses (Fig. 1d) while advancing LRP8-targeted candidates through postexposure treatment models and structure-guided optimization. These efforts will determine whether LRP8 inhibition can evolve from a protective strategy to a therapeutic solution for established TBEV infection. Strategies such as decoy receptor-based antiviral drugs are urgently needed to address emerging orthoflavivirus threats.^5^

## References

[1] Mittler, E. et al. LRP8 is a receptor for tick-borne encephalitis virus. *Nature* **646**, 945–952 (2025). 40993380 10.1038/s41586-025-09500-2 PMC13221092

[2] Li, P. et al. LRP8 is an entry receptor for tick-borne encephalitis viruses. *Proc. Natl Acad. Sci.* **122**, e2525771122 (2025). 41166431 10.1073/pnas.2525771122 PMC12595491

[3] Lindquist, L. & Vapalahti, O. Tick-borne encephalitis. *Lancet (Lond., Engl.)* **371**, 1861–1871 (2008). 10.1016/S0140-6736(08)60800-4 18514730

[4] Sui, L. et al. Multi-protomics analysis identified host cellular pathways perturbed by tick-borne encephalitis virus infection. *Nat. Commun.* **15**, 10435 (2024). 39616195 10.1038/s41467-024-54628-w PMC11608235

[5] Pierson, T. C. & Diamond, M. S. The continued threat of emerging flaviviruses. *Nat. Microbiol.* **5**, 796–812 (2020). 32367055 10.1038/s41564-020-0714-0 PMC7696730

